# The empirical basis for modelling glacial erosion rates

**DOI:** 10.1038/s41467-020-14583-8

**Published:** 2020-02-06

**Authors:** Simon J. Cook, Darrel A. Swift, Martin P. Kirkbride, Peter G. Knight, Richard I. Waller

**Affiliations:** 1 Geography and Environmental Science, School of Social Sciences, University of Dundee, Nethergate, Dundee, DD1 4HN UK; 2 Department of Geography, University of Sheffield, Winter Street, Sheffield, S10 2TN UK; 3 School of Geography, Geology and the Environment, Keele University, Staffordshire, ST5 5BG UK

**Keywords:** Cryospheric science, Geomorphology

## Abstract

Glaciers are highly effective agents of erosion that have profoundly shaped Earth’s surface, but there is uncertainty about how glacial erosion should be parameterised in landscape evolution models. Glacial erosion rate is usually modelled as a function of glacier sliding velocity, but the empirical basis for this relationship is weak. In turn, climate is assumed to control sliding velocity and hence erosion, but this too lacks empirical scrutiny. Here, we present statistically robust relationships between erosion rates, sliding velocities, and climate from a global compilation of 38 glaciers. We show that sliding is positively and significantly correlated with erosion, and derive a relationship for use in erosion models. Our dataset further demonstrates that the most rapid erosion is achieved at temperate glaciers with high mean annual precipitation, which serve to promote rapid sliding. Precipitation has received little attention in glacial erosion studies, but our data illustrate its importance.

## Abstract (web summary)

Glaciers have profoundly shaped Earth’s surface, but glacial erosion models lack a strong empirical basis. Cook et al. have compiled a dataset that illustrates how the speed at which glaciers move controls the rate at which they erode, and that climate is crucial in modulating glacier sliding speed and erosion rates.

## Introduction

Erosion by Quaternary glaciers and ice sheets has profoundly shaped large areas of Earth’s landscape^1–4^ and has been central to climate-tectonic feedbacks that influenced Earth’s climatic evolution during the late Cenozoic era^5–7^. Glacial erosion processes and rates are poorly understood because they operate in largely inaccessible and complex subglacial environments. The rate of shear at the ice-bed interface is widely agreed to be the most important control on erosion rates, but glacial erosion of bedrock is achieved via several distinct mechanisms, and theoretical treatments of these processes are constrained poorly by actual observations^1,8,9^. This complexity means that glacial landscape evolution models (LEMs) use a very simple erosion rule that relates glacial erosion rate (*E*) to glacier sliding velocity (*U*_*s*_), or surface velocity as its surrogate^4,7,9–16^. This rule is usually expressed as $E = K_GU_S^l$, where *K*_*G*_ is a bedrock erodibility constant and *l* is an exponent that is usually taken to be between one and two, although values of up to four have been used in LEMs depending on the value of *K*_*G*_ (e.g. ^1,4,8,13,14^). Rates and patterns of erosion in glaciated landscapes depend very sensitively on values of *K*_*G*_ and *l*, and the choice of *l* in particular has been a critical concern^8^.

Theoretical treatments of the main processes of glacial erosion, namely abrasion^9^ and quarrying^17,18^, provide qualified support for the so-called glacial erosion rule, but empirical support is limited. Values for *K*_*G*_ and *l* were first obtained empirically for Variegated Glacier, Alaska, where glacier surface velocity and sediment evacuation by subglacial drainage were monitored over a 2-year period that included an episode of active surge behaviour^19^. When augmented with data from two other temperate glaciers, this indicated a linear relationship between erosion and sliding speed (i.e. *l* ≈ 1)^19^. However, more recent studies that have determined erosion rates at tidewater glaciers^20^ and for a single alpine glacier^8^ have indicated a non-linear relationship (i.e. *l* ≥ 2). Hence, there is uncertainty about which value of *l* should be used in LEMs. Further, it is unclear to what extent erosion rule parameters derived from studies of surging glaciers^19^ can be considered representative of most glaciers globally, and hence of broad applicability in LEMs^21^. Surge behaviour is associated with dramatic changes in sliding and drainage system behaviour that are atypical of the great majority of glaciers.

**Fig. 1:**
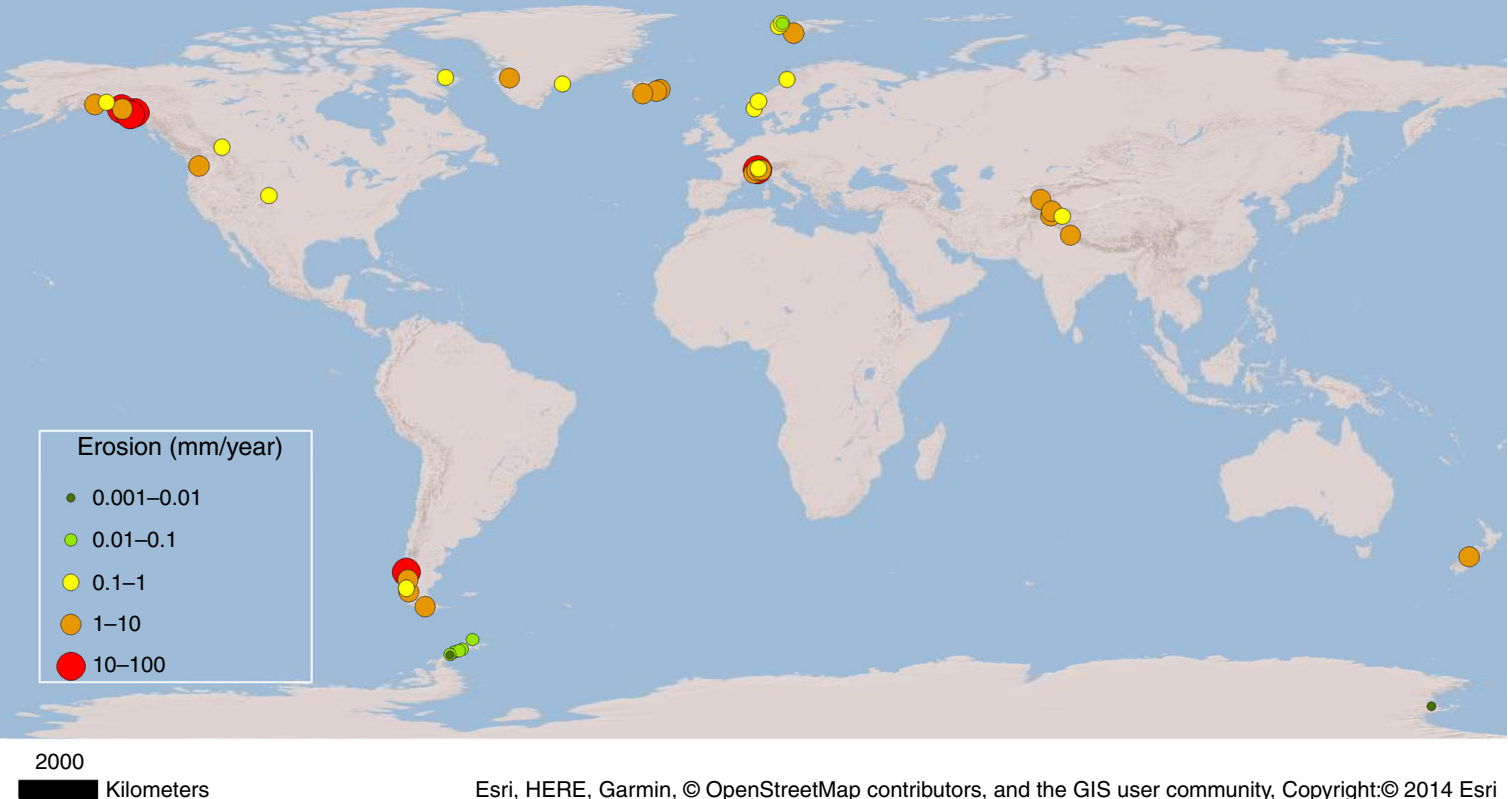
Map of sites used to compile glacial erosion rate data. Map of the 38 sites used to compile glacial erosion rate data, alongside data from Antarctic and Patagonian tidewater glaciers^20^. Details of study glaciers and data collection methods are contained in Supplementary Table 1.

Given the importance of the erosion rule for modelling landscape evolution and for understanding the glacial contribution of fine sediment production to global weathering budgets throughout the Quaternary, we constrain *l* using a new compilation of erosion rates and glacier sliding velocities for a dataset of 38 glaciers (Supplementary Table 1). As for previous studies, lack of access to the subglacial environment means that our study must rely mostly on published measurements of ice surface velocity and subglacial sediment evacuation. Our global dataset encompasses a wide range of climatic and geological environments (Fig. 1), and provides a robust empirical context within which to examine both glacial and non-glacial controls on glacial erosion. We use these data to examine the strength of the relationship between glacial erosion rate and glacier sliding velocity, enabling us to determine a value for the exponent *l* that allows models of glacial landscape evolution to be more reliably based on real-world relationships.

Intuitively, climate should be a strong control on glacial erosion because sliding is influenced by thermal regime and melt availability, yet empirical evidence for a relationship with climate is surprisingly limited^20^. A recent study^20^ selected glaciers on a latitudinal profile to isolate latitude as a climatic (i.e. temperature) proxy, and concluded that climate and glacier thermal regime were more significant factors than sliding in determining erosion rates. This conclusion was supported by data on mean annual air temperatures (MAAT), which indicated that erosion rates were higher for temperate glaciers than for Polar glaciers. However, the potentially important role of precipitation in controlling erosion rates has yet to be explored with an empirical dataset. We hypothesise that higher precipitation rates would lead to greater erosion rates because liquid precipitation reaching the glacier bed will enhance sliding and sediment flushing^22^, and higher rates of snowfall in the accumulation zone will result in thicker ice and steeper mass balance gradients, which should also lead to greater rates of sliding^23^. We use our glacial erosion dataset to examine how latitude, temperature and precipitation influence erosion rate, which may have important implications for modelling landscapes of glacial erosion and the development of erosion rules.

## Results

### Glacier sliding velocity and erosion rate

**Fig. 2:**
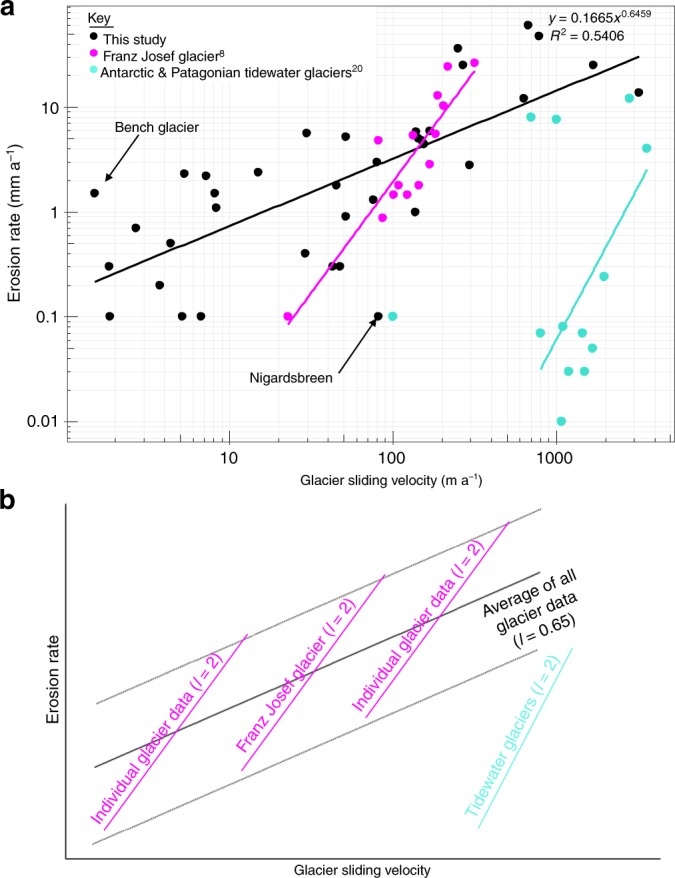
Plots illustrating the relationship between glacier sliding velocity and glacial erosion rate. **a** Our global dataset of glacier velocity against erosion rate with outliers indicated. The data are compared to data from the Franz Josef Glacier^8^ and a range of tidewater glaciers from the Antarctic Peninsula and Patagonia^20^. **b** Conceptual diagram illustrating that data for individual glaciers plot on a steeper gradient, but the global dataset, encompassing a variety of glacier contexts, plots on a shallower slope.

Our dataset shows variation in glacier erosion rates over five orders of magnitude (Fig. 1; Supplementary Table 1) and further shows that erosion rates vary by up to a factor of 100 for any given value of sliding velocity (Fig. 2a). Nonetheless, glacier erosion rates and sliding velocity are shown to be positively correlated (Fig. 2a: *R*^2^ = 0.54, *p* < 0.01), indicating a moderately strong and statistically significant association. From this relationship, we find *l* to be 0.65. A datapoint from Meserve Glacier (Wright Valley, Antarctica) is excluded from Fig. 2a, but represents a case of both extremely low sliding velocity and erosion rate (Supplementary Table 1). When Meserve Glacier is included in the regression analysis, the *R*^2^ value increases to 0.67 (*p* < 0.01), and *l* is 0.69. In either case, the dimensionless erosion rate factor, *K*_*G*_, is 1 × 10^−4^ (when erosion rate is expressed in m a^−1^).

### Climate and erosion rate

**Fig. 3:**
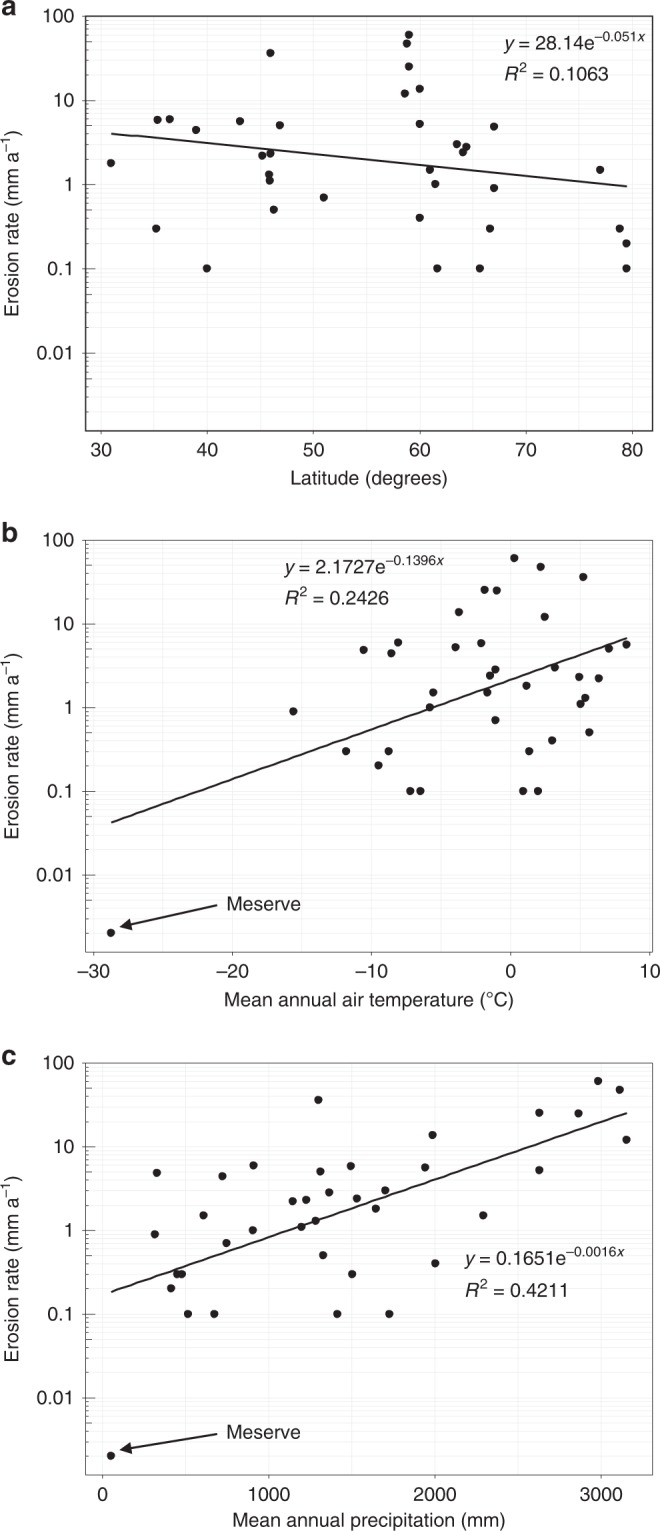
Plots illustrating the relationship between climate and glacial erosion rate. Glacial erosion rate plotted as a function of **a** latitude (north and south of the Equator); **b** mean annual air temperature (MAAT) and; **c** mean annual precipitation (MAP).

Latitude represents a proxy for ambient temperature conditions, which in turn control glacier thermal regime, and hence may control glacial erosion rates^20^. Nonetheless, our dataset shows that latitude has a weak and insignificant relationship (*R*^2^ = −0.11; *p* = 0.82) with glacial erosion rates (Fig. 3a). Thus, in order to investigate the potential for more direct relationships between specific climate variables and erosion rates, we used ERA-I reanalysis data for 1979–2000 in order to derive MAAT and MAP (mean annual precipitation) values (Fig. 3b, c; Supplementary Table 1) for each of the 38 glaciers in our dataset. This analysis shows a weak positive relationship between erosion rate and temperature (*R*^2^ = 0.24; *p* = < 0.01), although when the Meserve Glacier outlier is removed, the relationship weakens significantly (*R*^2^ = 0.06; *p* = 0.16). The relationship between erosion rate and precipitation is much stronger and significant, both including the Meserve Glacier outlier (*R*^2^ = 0.42; *p* = < 0.01), and excluding it (*R*^2^ = 0.38; *p* = < 0.01).

**Fig. 4:**
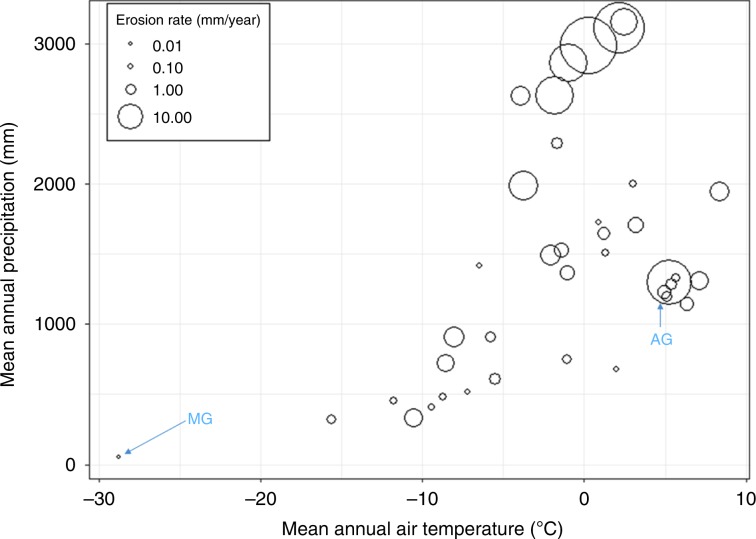
Plot of mean annual air temperature (MAAT) against mean annual precipitation (MAP) with bubble size reflecting corresponding erosion rate. MG Meserve Glacier, AG Argentière Glacier.

Investigation of the combined effect of MAAT and MAP values on erosion rates (Fig. 4) reveals a pattern of high erosion rates (>10 mm a^−1^) when MAAT values exceed around −2 °C and MAP values exceed around 2500 mm. However, glaciers exhibiting such rates are exclusively Alaskan, with one notable exception (Argentière Glacier).

## Discussion

The wide variation in erosion rates for glaciers of similar catchment size or sliding velocity illustrated from our dataset (Figs. 1, 2a) is evident in previous compilations^23^. This likely reflects variability in climate and lithology, which together influence ice thickness and sliding speed, sediment evacuation rate, and bedrock erodibility, as well as the wide range of measurement scales and methods (Supplementary Table 1)^24,25^. There is also a remarkable degree of overlap between our compiled dataset and the data from Franz Josef Glacier^8^, and from several Patagonian glaciers^20^ (Fig. 2; Supplementary Table 2). Further, we find that the mean dimensionless erosion rate factor, *K*_*G*_, from our dataset is 1 × 10^−4^, which is the same as that indicated by most previous field estimates^8,19^, and is the value used most commonly in LEM studies (e.g. see refs. ^4,7,13,14^). An important finding is that the value of *l* indicated by our dataset is lower than the value of 1 that is typically assumed in glacial erosion models (based on data from a single surge-type glacier^19^), and much lower than empirically derived maximum estimates of 2.02^8^ and 2.34–2.62^20^ that have been obtained for individual glaciers.

A value of *l* that is <1 implies a rate of increase in erosion that is lower than the rate of increase in sliding. This finding is consistent with hard-bed sliding theories that predict a decrease in ice-bed contact as greater sliding speeds cause the size of cavities formed in the lee of bedrock obstacles to increase^26^. A similar negative feedback is evident in certain theoretical treatments of quarrying^18^, which also find that the value of *l* should be <1. Nonetheless, the value of *l* from our dataset differs greatly from that obtained using robust, longitudinally distributed measurements of sliding and bedrock erosion at the non-surging Franz Josef Glacier^8^ (i.e. *l* ≈ 2). We note, however, that (1) the data from Franz Josef Glacier lie within the broad envelope of our data set (Fig. 2a; Supplementary Table 2), and (2) our study employs a different approach whereby we have used single sliding velocity values and average erosion rate estimates for each glacier in the dataset. We believe that the latter difference critically affects estimation of *l* because the mechanics of sliding and erosion are anticipated to vary along the typical glacier longitudinal profile, yielding a non-linear relationship between sliding and erosion rate. Specifically, it is likely that conditions for erosion are optimised in the upper ablation area, just below the ELA, where ice is thick and seasonal surface melt forms subglacial channels that flush sediment whilst also promoting diurnally high basal water pressures that stimulate the highest sliding rates^8,12,25,27^. Erosion rates elsewhere along the glacier profile should be much lower. In the accumulation zone, low volumes of surface melt should reduce sliding and sediment flushing rates^28^. In the ablation area generally, sediment flushing and erosion are greater, but in the lower ablation zone, thinner ice and lower mean basal water pressures, due to the increased efficiency of subglacial channels, yields lower sliding velocities, thus moderating the rate of erosion^25,27^. Hence, sliding-erosion rate data for individual glaciers should typically plot on a steeper, non-linear gradient than that defined by spatially averaged values from many glacial catchments, which integrate the longitudinal variations envisaged here. Thus, our study is consistent with studies of individual glaciers which yield higher values of *l*. These relationships are summarised in Fig. 2b.

Individual glaciers that do not fit our relationship well include Nigardsdreen (Norway) and Bench Glacier (Alaska) (Fig. 2a). Data from these glaciers point to controls on erosion beyond sliding rate that are exerted by lithology, hydrology and climate. Nigardsbreen demonstrates an unusually low erosion rate, which may be related to its granite-gneiss substrate with low erodibility. Bench Glacier, in contrast, demonstrates rapid erosion for a small glacier, which may be due to the ability of many small glaciers to rapidly develop efficient, seasonal subglacial drainage systems that rapidly evacuate erosion products^25,27^ and thereby enhance ice-bed contact. Meserve Glacier (Antarctic Dry Valleys; not shown in Fig. 2a) fits the global trend well, but is an extreme data point compared to the bulk of the dataset. Meserve Glacier achieves very low erosion rates as a consequence of its location in a polar desert environment where basal melt and, therefore, sliding are negligible^29^.

Another set of data that is a particularly poor fit with our compilation is that for tidewater glaciers on the Antarctic Peninsula^20^ (Fig. 2a; Supplementary Table 2), where rapid ice flow is associated with very low erosion rates. The pattern of data for Antarctic Peninsula glaciers also differs markedly from that for Franz Josef Glacier^8^, and for Patagonian tidewater glaciers^20^ (Fig. 2; Supplementary Table 2). Antarctic tidewater environments appear, therefore, to represent a special context that may require careful consideration for landscape evolution modellers. It has been proposed^20^ that these low erosion rates are a consequence of a lack of surface meltwater reaching the bed, thereby reducing basal water pressure fluctuations that are important for subglacial quarrying, as well as sediment flushing rates. To yield insights into the glacier erosion rule that are applicable to the majority of glaciers globally, it is therefore necessary to exclude Antarctic tidewater glaciers from our analysis. The reported sliding-erosion relationship^20^ that is specific to these glaciers nonetheless appears robust, and valid for models that consider this specific context.

Previous research^20^ has shown a correlation between latitude, which serves as a proxy for climate (i.e. temperature), and glacial erosion rates. This work isolated latitude as a control on glacial erosion by selecting tidewater glaciers only along a latitudinal gradient from Antarctica through Patagonia. These data illustrated the importance of climate as an underlying control on glacier thermal regime, and hence ice dynamics and erosion. However, our dataset, which comprises a larger sample of glaciers across a wider latitudinal range, shows an insignificant relationship between glacial erosion and latitude (Fig. 3a). We conclude that latitude alone is an insufficient proxy for climate for use in numerical ice-erosion or landscape evolution models, unless its influence can be isolated from that of other variables. Hence, erosion rate is better predicted by directly observed or calculated glacier sliding velocities. This does not, however, preclude the existence of direct associations between specific climate variables and glacier erosion rates.

We find that there is a weak relationship between temperature and erosion rates only if the data point from Meserve Glacier, which represents extremely cold temperature and low erosion rate, is included (Fig. 3b). Otherwise, there does not appear to be a systematic increase in erosion rate with increasing MAAT. Conversely, there is a positive relationship between precipitation and glacial erosion rate (Fig. 3c), which has hitherto not been demonstrated empirically^30^. High precipitation may be associated with more erosive glaciers because wet precipitation at low altitudes is observed to enhance sliding^8,27^, whilst solid precipitation at higher altitudes enhances sliding by steepening the mass balance gradient. These results are consistent with the suggested correspondence of Quaternary erosion rate maxima with precipitation maxima because higher precipitation translates into higher ice fluxes and sliding velocities^30^.

Taken together, both temperature and precipitation appear to have a strong influence on glacial erosion. Figure 4 shows that the greatest rates of glacial erosion are achieved when MAAT exceeds ~ −2 °C, which represents mostly temperate regimes, and when MAP exceeds ~2500 mm. Alaskan tidewater glaciers in particular fall into this category. Further, rapid plate convergence and tectonic uplift mean Alaskan glaciers are situated on highly fractured bedrock, which is likely to facilitate very rapid bedrock quarrying rates^23^, and are likely to possess steeper gradients that promote faster sliding^31^. A notable outlier in the MAAT-MAP dataset is the Argentière Glacier (AG in Fig. 4^32^). This is one of the glacier data points used in the first attempt to derive the key parameters of the glacial erosion rule^19^. This published erosion rate was calculated using the maximum (rather than mean) observed depth of erosion by ice into a small marble plate that was installed at the glacier bed, and is thus likely to be an overestimate of glacier-scale erosion.

Overall, we have compiled a new dataset to show that glacier sliding rate and climate are key determinants of glacial erosion rate. These results have important implications for the integration of glacial erosion into LEMs, and yield new insights into the underlying controls on glacial erosion. Analysis of our global dataset indicates that a value of *l* ≤ 1 (~0.65) is appropriate for use in glacial erosion models where the goal is to understand the glacial contribution to overall erosion and sedimentation rates at the landscape scale (i.e. across large ice sheet domains or multiple glaciated catchments). A value of *l* ≥ 2 is appropriate and empirically justified^8^, however, where the goal is to simulate the long profile evolution of individual glacial valleys because it captures along-profile changes in sliding and erosion mechanics.

Further, our results confirm the importance of climate as an underlying control on glacial erosion^20^, but for the first time illustrate the hitherto underappreciated role of precipitation in driving glacial erosion, and that glacial erosion is influenced by both temperature and precipitation thresholds. Nonetheless, ours remains a relatively small dataset wherein the highest erosion rates, which coincide with temperate and high precipitation conditions, are those associated with Alaskan glaciers, which may also be influenced by rapid rock uplift rates. We suggest that greater community effort is required to obtain a larger global dataset of glacial erosion rates if climate parameter relationships are to be found that can replace direct or calculated measurements of ice sliding velocity in glacial landscape and Earth System models.

## Methods

### Erosion rate data

Glacial erosion rate data have been compiled in previous studies^23,33^. We used many of the modern erosion rate data listed in these studies, and, on the basis of an extensive literature review, supplemented it with additional erosion rate values cited in more recent studies. The full compiled list of data used in this study is shown in Supplementary Table 1. It was not possible to use all of the modern erosion rate data cited in these previous compilations, particularly where there were no reliable corresponding velocity values available (see below). This was the case for some large glaciers, surge-type glaciers, and where erosion rates were derived from glaciated catchments, rather than from individual glaciers. We have reported mean values of erosion rate for sites where multiple erosion rate estimates were available. Most of the erosion data are derived from the gauging of meltwater streams, although a number of studies derived values from marine, lacustrine or proglacial sediment accumulations, and two of the studies derived values from the study of englacial sediments.

Most of the data points in Supplementary Table 1 are from previous compilations of erosion rate data where uncertainties have already been estimated^23,33^. Uncertainty in erosion rates derived from detailed, multi-year studies of sediment export in proglacial meltwater streams has previously been estimated to be 10%; for glaciers where the meltwater gauging records are shorter, or where it was difficult to estimate sediment density due to catchment lithological variability, the uncertainty in erosion rate estimates is considered to be as much as 50%^23^. A more recent study^20^ of erosion rates derived from marine sediment accumulations in front of Antarctic and Patagonian tidewater glaciers similarly estimated uncertainties to be between 38 and 50%. These uncertainties should be considered within the context of the multi-order-of-magnitude variation in glacial erosion rate estimates (Fig. 1, Supplementary Table 1).

### Glacier sliding velocity

Glacier velocity data were obtained from published sources (Supplementary Table 1). Total glacier velocity is the sum of sliding and internal deformation. Sliding is most relevant in terms of glacial erosion, although previous studies that have examined the velocity-erosion relationship have variously used measured sliding velocity^19,32^, surface velocity as a surrogate for sliding velocity^8,19^, or sliding velocity modelled from surface velocity^20^. Wherever possible, we used velocity values that were a direct measure of sliding velocity, but we have calculated most of the sliding velocity (*U*_s_) values shown in Supplementary Table 1 from published surface velocity (*U*_surf_) values according to:

1 $$U_{\mathrm{s}} = U_{\mathrm{surf}} - \frac{{2A}}{{n + 2}}\left( {\rho g\mathrm{sin}\alpha } \right)^n\,h^{n + 1}$$

where *A* is a temperature-dependent ice softness parameter (*A* = 2.4 × 10^24^ s^−1^ Pa^−3^ for temperate ice; *A* = 1.7 × 10^24^ s^−1^ Pa^−3^ for cold ice), n is an exponent, usually taken to be 3, *ρ* is ice density, *g* is gravitational acceleration, *α* is ice surface slope, and h is ice thickness (in metres). *U*_surf_ values were generally taken from the ELA (equilibrium line altitude), in the middle reaches of the glacier (i.e. down-glacier from the ELA), or, in the case of calving glaciers, near the calving front. These generally represent the upper end of ice velocities for our sampled glaciers. This is consistent with previous compilations of erosion and velocity data where velocities have been measured at the ELA^20^, the middle reaches of the glacier^19^, and at the terminus;^32^ data from the Franz Josef Glacier show peak velocities down-glacier from the ELA^8^. Velocity values that were contemporaneous with the collection of erosion rate data were preferred, but matching velocities with erosion rates in this way was not always possible. Wherever possible, sliding velocity was calculated using ice thickness measured at the point where glacier velocity was quantified. Where this was not possible, a global dataset of modelled ice thicknesses was used instead^34^ to provide a thickness estimate at the point of velocity measurement. Glacier surface slope was measured from Google Earth across the point of velocity measurement, unless surface slope was stated in the study where velocity had been measured.

Our velocity dataset is derived from a combination of studies of multi-year observations of velocity, which yield more robust estimates of average velocity conditions, and of studies where velocities were measured over a shorter time period. In the latter case, these shorter-term observations were made in the summer when glaciers may undergo significant dynamic changes in response to the evolution of the subglacial hydrological system. This represents a degree of uncertainty in the velocity data for those glaciers, which could represent periods of speed-up or slow-down depending on the nature of the hydrological network at the time of measurement, and may differ from winter velocities. Nonetheless, previous erosion rate-velocity rule parameterisations have been developed for summertime velocity conditions^8,20^.

### Climate data

MAAT and MAP data were derived from ERA-Interim (ERA-I) datasets, which can be downloaded free-of-charge from the European Centre for Medium-Range Weather Forecasts (ECMWF^35^) . The spatial resolution of the data is ~20 km × 20 km on 60 vertical levels, and it has been employed previously in global analyses of glacial climate characteristics^36^. Data on ‘2 m temperature’ and ‘total precipitation’ were downloaded in.netcdf format for the period January 1979 to December 2000, which overlaps, at least in part, with the time period over which most of the erosion rate data were collected. These were loaded into ArcGIS 10.5 using the ‘Make NETCDF Raster Layer’ tool. The Raster Calculator was used to compute mean values of temperature and precipitation from 1979 to 2000, and the climatic values were extracted for each glacier.

The time difference between the climate data census period and the time windows represented by some of the datapoints is a source of uncertainty, particularly for erosion rate data averaged over the last few hundred years—a period when average climate may have been cooler than for 1979–2000. Further, the spatial resolution of the dataset means that climate conditions are averaged over variable topography and environmental conditions (e.g. coast to mountains in Alaska). Despite these uncertainties, it is considered here that the homogenous dataset is an advantage in drawing out global patterns of climate and its links with glacial erosion rates^36^, and alternative climate data, particularly representing the last few hundred years, may also have large uncertainties.

## Supplementary information

Supplementary Information

## Data Availability

All data generated or analysed during this study are included in this article (and its Supplementary Information files).

## Code availability

All statistical analyses were undertaken in RStudio, and Figs. 2 to 4 were drawn using the ggplot2 package^37^. The code is available from the corresponding author, S.J.C., on request.
