# Supplementary material for: The empirical basis for modelling glacial erosion rates
Source: Nat Commun. 2020 Feb 6;11:759. doi: 10.1038/s41467-020-14583-8 (PMC7005307; doi:10.1038/s41467-020-14583-8)
Supplement: Supplementary file 1 — Supplementary Information [file 41467_2020_14583_MOESM1_ESM.pdf]

Supplementary Materials

Supplementary Table 1: *Glacial erosion, sliding velocity and climate data. MAAT is Mean Annual Air Temperature, and MAP is Mean Annual Precipitation, both derived from ERA-Interim data.*

**Location**	**Latitude**	**Dynamic context**	**Erosion rate (mm a^{-1})**	**Sliding velocity (m a^{-1})**	**MAAT (°C)**	**MAP (mm)**	**Nature and source of erosion data**	**Nature and source of velocity data**
Alaska								
Bench	61	Land-terminating	1.50	1.5	-1.7	2293.0	Meltwater gauging from 1999-2002[1]	Sliding velocity from 1999-2002 glacier-wide velocity study[1]
Crillon	58.6	Tidewater	12.01	636.9	2.5	3157.1	Marine sediments from 1926-1961[2]	Modelled sliding velocity from 1983-1987 surface velocity near calving terminus[3]<br>Surface velocity: 640 m a^{-1}<br>Ice thickness: 160 m[(4)]<br>Surface slope: 0.06 m/m
Grand Pacific	59	Tidewater	24.86	268.6	-1.0	2867.3	Marine sediments (unknown time period); Average of values from[2]	Modelled sliding velocity from 1988-1991 surface velocity near calving terminus[5]<br>Surface velocity: 269 m a^{-1}<br>Ice thickness: 120 m[(4)]<br>Surface slope: 0.05 m/m
Hubbard	60	Tidewater	13.71	3199.5	-3.7	1990.7	Marine sediments from last 700 years[2]	Modelled sliding velocity from 1988-1989 surface velocity near calving terminus[6]<br>Surface velocity: 3200 m a^{-1}<br>Ice thickness: 150 m[(4)]<br>Surface slope: 0.04 m/m
Johns Hopkins	58.8	Tidewater	47.24	787.1	2.2	3114.3	Marine sediments (unknown time period)[2]	Modelled sliding velocity from 1983-1987 surface velocity near calving terminus[3]<br>Surface velocity: 800 m a^{-1}<br>Ice thickness: 200 m[(4)]<br>Surface slope: 0.07 m/m
Kennicott	61.5	Land-terminating	1.00	138.0	-5.8	909.5	Meltwater gauging from 1999 & 2000; Average of values from[7]	Modelled sliding velocity from 2000 surface velocity of lower glacier[8]<br>Surface velocity: 146 m a^{-1}<br>Ice thickness: 350 m[(8,9)]<br>Surface slope: 0.03 m/m
Margerie	59	Tidewater	60.07	672.7	0.3	2986.5	Meltwater gauging (unknown time period; ~1994?)[2]	Modelled sliding velocity from 1988-1991 surface velocity near calving terminus[5]<br>Surface velocity: 679 m a^{-1}<br>Ice thickness: 200 m[(4)]<br>Surface slope: 0.06 m/m
Muir	59	Tidewater	25.10	1697.5	-1.8	2632.7	Marine sediments representing ~2100 years in early Holocene; Average of values from[2]	Modelled sliding velocity from 1988-1991 surface velocity near calving terminus[5]<br>Surface velocity: 1700 m a^{-1}<br>Ice thickness: 100 m[(4)]<br>Surface slope: 0.10 m/m

Variegated	60	Land-terminating	5.19	51.9	-3.9	2631.8	Meltwater gauging from 1982-1984[10]	Sliding velocity assumed from 1982-1984 surface velocity of upper glacier[10]
European Alps								
Argentiere	46.0	Land-terminating	36.00	250.0	5.3	1300.5	Direct measurement from subglacial experiments[11]	Unclear; assumed to be measured sliding velocity[11]
Bossons	45.9	Land-terminating	1.28	76.5	5.4	1286.0	Meltwater gauging (unknown time period); Average of values from[12]	Modelled sliding velocity from 1958 surface velocity close to ELA[13]<br>Surface velocity: 310 m a^{-1}<br>Ice thickness: 134 m[(4)]<br>Surface slope: 0.32 m/m
Gornergletscher	45.9	Land-terminating	1.11	8.4	5.1	1200.7	Meltwater gauging from 1970-1980; Average of values from[2]	Modelled sliding velocity from 2005 surface velocity of lower glacier[14]<br>Surface velocity: 24 m a^{-1}<br>Ice thickness: 250 m[(14)]<br>Surface slope: 0.05 m/m
Haut Glacier d'Arolla	45.95	Land-terminating	2.26	5.3	5.0	1230.1	Meltwater gauging from 1989-1990; Average of values from[7]	Modelled sliding velocity from 1995-1996 surface velocity of lower glacier[15]<br>Surface velocity: 10 m a^{-1}<br>Ice thickness: 103 m[(15)]<br>Surface slope: 0.12 m/m
St Sorlin	45.2	Land-terminating	2.20	7.2	6.4	1146.4	Meltwater gauging (unknown time period)[12]	Modelled sliding velocity from 1958-1967 glacier-wide velocity study[16]<br>Surface velocity: 8 m a^{-1}<br>Ice thickness: 50 m[(4)]<br>Surface slope: 0.18 m/m
Tsanfleuron	46.3	Land-terminating	0.50	4.4	5.7	1331.4	Englacial sediment samples collected from across glacier in 2007[(17)]	Sliding velocity from 2004-2006 borehole survey of lower glacier[18]
Central Asia								
Batura	36.5	Land-terminating	5.85	169.0	-8.1	911.9	Meltwater gauging during 1990 ablation season; Average of values from[12]	Modelled sliding velocity from 1974-1975 surface velocity in middle reaches of glacier[19]<br>Surface velocity: 170 m a^{-1}<br>Ice thickness: 200 m[(4)]<br>Surface slope: 0.03 m/m
Fedchenko	39	Land-terminating	4.36	155.4	-8.6	724.9	Meltwater gauging from 1925-1959[2]	Modelled sliding velocity from 1958 surface velocity in middle reaches of glacier[20]<br>Surface velocity: 263 m a^{-1}<br>Ice thickness: 582 m[(20)]<br>Surface slope: 0.03 m/m
Gangotri	30.9	Land-terminating	1.80	45.4	1.2	1648.5	Meltwater gauging from 2000-2003[(12)]	Modelled sliding velocity from 2007-8 surface velocity close to ELA[21]<br>Surface velocity: 47 m a^{-1}<br>Ice thickness: 210 m[(4)]<br>Surface slope: 0.03 m/m
Raikot	35.4	Land-terminating	5.75	138.9	-2.1	1495.8	Meltwater gauging (unknown time period)[12]	Modelled sliding velocity from 1954 surface velocity in middle reaches of glacier[22]

								Surface velocity: 178 m a^{-1}<br>Ice thickness: 235 m[(4)]<br>Surface slope: 0.08 m/m
Siachen	35.3	Land-terminating	0.25	43.0	-11.8	452.19	Meltwater gauging from 1986-1991[(12)]	Modelled sliding velocity from 2008-2009 glacier-wide surface velocity study[23]<br>Surface velocity: 44.9 m a^{-1}<br>Ice thickness: 235 m[(4)]<br>Surface slope: 0.03 m/m
Greenland								
Leverett	67	Land-terminating	4.80	150.3	-10.6	332.0	Meltwater gauging from 2009-2010[(24)]	Modelled sliding velocity from 2010 surface velocity of lower glacier[25]<br>Surface velocity: 155 m a^{-1}<br>Ice thickness: 405 m[(24)]<br>Surface slope: 0.02 m/m
Mittivakat	65.7	Land-terminating	0.13	6.7	-6.5	1416.6	Proglacial lake sediments from 1970-1994[(12)]	Modelled sliding velocity from 1996-2011 glacier-wide surface velocity study[26]<br>Surface velocity: 8 m a^{-1}<br>Ice thickness: 235 m[(4)]<br>Surface slope: 0.09 m/m
Norway								
Bondhusbreen	60	Land-terminating	0.44	29.2	3.0	2004.6	Meltwater gauging from 1968-1986[(2)]	Sliding velocity from 1980-1982 subglacial tunnel instrumentation study[27]
Engabreen	66.7	Land-terminating	0.27	47.5	1.3	1507.4	Meltwater gauging from 1968-1986; Average of values from[2,12,28]	Basal ice velocity from 1996-1997 subglacial tunnel instrumentation study[29]
Nigardsbreen	61.7	Land-terminating	0.13	81.7	0.9	1727.9	Meltwater gauging from 1968-1986; Average of values from[2,12,28]	Modelled sliding velocity from 1968-1969 surface velocity of middle reaches of glacier[30]<br>Surface velocity: 220 m a^{-1}<br>Ice thickness: 200 m[(30)]<br>Surface slope: 0.16 m/m
Svalbard								
Broggerbreen	78.9	Land-terminating	0.27	1.8	-8.7	479.6	Meltwater gauging (unknown time period)[2]	Modelled sliding velocity from 1970-1988 surface velocity close to ELA[31]<br>Surface velocity: 2 m a^{-1}<br>Ice thickness: 100 m[(32)]<br>Surface slope: 0.04 m/m
Erikbreen	79.5	Lake-terminating	0.19	3.8	-9.5	414.5	Meltwater gauging 1989-1992[(2)]	Sliding velocity from 1990-1991 glacier-wide velocity study[32]
Finsterwalderbreen	77	Land-terminating	1.45	8.2	-5.5	609.9	Meltwater gauging from 1999-2000; Average of values from[33]	Modelled sliding velocity from 1950-1952 surface velocity close to ELA[32]<br>Surface velocity: 14.3 m a^{-1}<br>Ice thickness: 260 m[(32)]<br>Surface slope: 0.04 m/m

Hannabreen	79.5	Land-terminating	0.08	1.9	-7.2	517.6	Meltwater gauging 1992(2)	Sliding velocity assumed to be ~0.3 times the 1990-1991 surface velocity of lower glacier based on similar observations at Erikbreen[32]<br>Surface velocity: 6.2 m a^{-1}
Western USA & Canada								
Arapaho	40	Land-terminating	0.13	5.2	2.0	678.1	Proglacial sediments (moraine, lake outlet) sampled during 1972 & 1973(34)	Modelled sliding velocity from 1972-1973 surface velocity close to ELA[34]<br>Surface velocity: 5.2 m a^{-1}<br>Ice thickness: 11 m(35)<br>Surface slope: 0.35 m/m
Nisqually	46.9	Land-terminating	5.00	144.0	7.1	1315.4	Meltwater gauging in 1976(36)	Sliding velocity from 1968-1970 surface velocity close to ELA[37]
Washmawupta	51	Land-terminating	0.70	2.7	-1.1	749.5	Meltwater gauging in 2007(38)	Sliding velocity from 2006-2008 glacier-wide surface velocity study[38,39]
Iceland								
Breiðamerkurjökull	64.1	Tidewater	2.41	15.0	-1.4	1533.8	Direct measurement; Average of values from[11]	Sliding velocity from subglacial instrumentation study[11]
Hoffellsjökull	64.4	Land-terminating	2.77	294.7	-1.1	1369.4	Meltwater gauging (unknown time period); Average of values from[2]	Modelled sliding velocity from 2002-2003 surface velocity of middle reaches of glacier[40]<br>Surface velocity: 360 m a^{-1}<br>Ice thickness: 300 m(4)<br>Surface slope: 0.07 m/m
Jökulsa	63.5	Land-terminating	3.00	80.0	3.2	1703.7	Meltwater gauging from 1988-1990; Average of values from[2,12,28]	Sliding velocity below ELA from glacier flow modelling study[41]
Arctic Canada								
Coronation	67	Tidewater	0.86	51.5	-15.6	320.4	Marine sediments representing ~2100 years in early Holocene;[2]	Modelled sliding velocity from 2011-2012 glacier-wide surface velocity study[42]<br>Surface velocity: 52.1 m a^{-1}<br>Ice thickness: 301 m(4)<br>Surface slope: 0.01 m/m
New Zealand								
Ivory	43.1	Lake-terminating	5.60	29.7	8.6	1943.5	Proglacial lake sediments from 1976-1985(2)	Modelled sliding velocity from 1986(?) surface velocity near calving terminus[43]<br>Surface velocity: 30 m a^{-1}<br>Ice thickness: 50 m(4)<br>Surface slope: 0.13 m/m
Antarctica								

Meserve	77.5	Land-terminating	2.00×10^{-3}	8.0×10^{-3}	-28.7	53.7	Englacial sediment sampled in 1995-1996[(44)]	Sliding velocity from 1995-1996 subglacial instrumentation study[45]

Supplementary Table 2: *Regression relationships of glacier sliding velocity against erosion rate for our dataset combined with averaged data from the Franz Josef Glacier[46] and data from Patagonian and Antarctic tidewater glaciers[47].*

**Dataset**	**Number of datapoints**	**Exponent (*l*)**	**Correlation coefficient (r^2)**	**p-value**
Our data	38	0.69	0.67	< 0.01
Our data excluding Meserve Glacier outlier	37	0.65	0.54	< 0.01
Our data including Franz Josef Glacier average[8]	39	0.70	0.67	< 0.01
Our data incuding Patagonian tidewater glaciers[20]	43	0.58	0.56	< 0.01
Our data incuding Franz Josef Glacier[8] and Patagonian tidewater glaciers[20]	44	0.58	0.56	< 0.01
Our data including Franz Josef Glacier[8], Patagonian and Antarctic tidewater glaciers[20]	52	0.28	0.10	0.02

1 Riihimaki, C. A., MacGregor, K. R., Anderson, R. S., Anderson, S. P. & Loso, M. G. Sediment evacuation and glacial erosion rates at a small alpine glacier. *J. Geophy. Res.: Earth Surf.* **110** (2005).

2 Hallet, B., Hunter, L. & Bogen, J. Rates of erosion and sediment evacuation by glaciers: A review of field data and their implications. *Glob. Plan. Change* **12**, 213-235 (1996).

3 Pelto, M. S. & Warren, C. R. Relationship between tidewater glacier calving velocity and water depth at the calving front. *Ann. Glac.* **15**, 115-118 (1991).

4 Farinotti, D. et al. A consensus estimate for the ice thickness distribution of all glaciers on Earth. *Nat. Geosci*. **12**, 168–173 (2019).

5 Hunter, L. E., Powell, R. D. & Lawson, D. E. Flux of debris transported by ice at three Alaskan tidewater glaciers. *J. Glac.* 42, 123-135 (1996).

6 Stearns, L. A., Hamilton, G. S., van der Veen, C. J., Finnegan, D. C., O'Neel, S., Scheick, J. B., and Lawson, D. E. Glaciological and marine geological controls on terminus dynamics of Hubbard Glacier, southeast Alaska. *J. Geophy. Res.: Earth Surf.* **120**, 1065– 1081 (2015).

7 Anderson, S. P. Glaciers show direct linkage between erosion rate and chemical weathering fluxes. *Geomorphology* **67**, 147-157 (2005).

8 Anderson, R. S., Walder, J.S., Anderson, S.P., Trabant, D.C. and Fountain, A.G. The dynamic response of Kennicott Glacier, Alaska, USA, to the Hidden Creek Lake outburst flood. *Ann. Glac.* **40**, 237-242 (2005).

9 Anderson, S. P., Walder, J.S., Anderson, R.S., Kraal, E.R., Cunico, M., Fountain, A.G. and Trabant, D.C. Integrated hydrologic and hydrochemical observations of Hidden Creek Lake jökulhlaups, Kennicott glacier, Alaska. *J. Geophys. Res.* **108** (2003).

10 Humphrey, N. F. & Raymond, C. F. Hydrology, erosion and sediment production in a surging glacier: Variegated Glacier, Alaska, 1982–83. *J. Glac.* **40**, 539-552 (1994).

11 Boulton, G. S. Processes and patterns of glacial erosion. In *Glacial Geomorphology*. (ed. D.R. Coates) 41-87 (Springer Netherlands), (1984)

12 Delmas, M., Calvet, M. & Gunnell, Y. Variability of Quaternary glacial erosion rates – A global perspective with special reference to the Eastern Pyrenees. *Quat. Sci. Rev*. **28**, 484-498 (2009).

13 Finsterwalder, R. Chamonix Glaciers. *J. Glac*. **3**, 547-548 (1959).

14 Sugiyama, S., Bauder, A., Riesen, P. & Funk, M. Surface ice motion deviating toward the margins during speed-up events at Gornergletscher, Switzerland. *J. Geophy. Res.: Earth Surf.*, **115** (2010).

15 Willis, I., Mair, D., Hubbard, B., Nienow, P., Fischer, U.H. and Hubbard, A. Seasonal variations in ice deformation and basal motion across the tongue of Haut Glacier d'Arolla, Switzerland. *Ann. Glac.* **36**, 157-167 (2003).

16 Vincent, C., Vallon, M., Reynaud, L. & Le Meur, E. Dynamic behaviour analysis of glacier de Saint Sorlin, France, from 40 years of observations, 1957–97. *J. Glac.* **46**, 499-506 (2000).

17 Roberson, S. *Characterization and quantifcation of glacial sediment transport in two small valley glaciers*, PhD Thesis, Aberystwyth University (2010).

18 Chandler, D., Hubbard, B., Hubbard, A., Murray, T. & Rippin, D. Optimising ice flow law parameters using borehole deformation measurements and numerical modelling. *Geophys. Res. Lett.* **35**, (2008).

19 Shroder, J.F.Jr., Saqib Khan R. J., M. & Spenser, M. Batura Glacier Terminus, 1984, Karakorum Himalaya. *Geol. Bull.(University of Peshawar)* **17**, 119-126 (1984).

20 Lambrecht, A., Mayer, C., Aizen, V., Floricioiu, D. & Surazakov, A. The evolution of Fedchenko glacier in the Pamir, Tajikistan, during the past eight decades. *J. Glac.* **60**, 233-244 (2014).

21 Satyabala, S. P. Spatiotemporal variations in surface velocity of the Gangotri glacier, Garhwal Himalaya, India: Study using synthetic aperture radar data. *Rem. Sens. Env.* **181**, 151-161 (2016).

22 Shroder, J. F., Bishop, M. P., Copland, L. & Sloan, V. F. Debris-covered Glaciers and Rock Glaciers in the Nanga Parbat Himalaya, Pakistan. *Geog. Ann.: Series A, Phys. Geog.* **82**, 17-31 (2000).

23 Agarwal, V. *et al.* Area and mass changes of Siachen Glacier (East Karakoram). *J. Glac.* **63**, 148-163, doi:10.1017/jog.2016.127 (2017).

24 Cowton, T., Nienow. P., Bartholomew, I., Sole, A., Mair, D. Rapid erosion beneath the Greenland ice sheet. *Geology* **40**, 343-346 (2012).

25 Cowton, T., Nienow, P., Sole, A., Wadham, J., Lis, G., Bartholomew, I., Mair, D., and Chandler, D. Evolution of drainage system morphology at a land-terminating Greenlandic outlet glacier. *J. Geophy. Res.: Earth Surf.,* **118**, 29-41 (2013).

26 Mernild, S. H. *et al.* Volume and velocity changes at Mittivakkat Gletscher, southeast Greenland. *J. Glac.*, **59**, 660-670 (2013).

27 Hagen, J. O., Wold, B., Liestøl, O., Østrem, G. & Sollid, J. L. Subglacial Processes at Bondhusbreen, Norway: Preliminary Results. *Ann. Glac.* **4**, 91-98 (1983).

28 Bogen, J. Erosion rates and sediment yields of glaciers. *Ann. Glac.* **22**, 48-52 (1996).

29 Cohen, D., Hooke, R. L., Iverson, N. R. & Kohler, J. Sliding of ice past an obstacle at Engabreen, Norway. *J. Glac.* **46**, 599-610 (2000).

30 Østrem, G., Liestøl, O. & Wold, B. Glaciological investigations at Nigardsbreen, Norway. *Norsk Geog. Tids.* 30, 187-209 (1976).

31 Hagen, J. O., Liestøl, O., Roland, E. & Jørgensen, T. Glacier atlas of Svalbard and Jan Mayen. (1993).

32 Etzelmuller, B., Vatne, G., Ødegård, R. S. & Sollid, J. L. Dynamics of Two Subpolar Valley Glaciers. Erikbreen and Hannabreen, Liefdefjorden, Northern Spitsbergen. *Geog. Ann.: Series A, Phys. Geog.*, **75**, 41-54 (1993).

33 Hodgkins, R., Cooper, R., Wadham, J. & Tranter, M. Suspended sediment fluxes in a high-Arctic glacierised catchment: implications for fluvial sediment storage. *Sed. Geol.* **162**, 105-117 (2003).

34 Reheis, M. J. Source, Transportation and Deposition of Debris on Arapaho Glacier, Front Range, Colorado, U.S.A. *J. Glac.* **14**, 407-420 (1975).

35 Haugen, B. D., Scambos, T.A., Pfeffer, W.T. and Anderson, R.S. Twentieth-century changes in the thickness and extent of Arapaho Glacier, Front Range, Colorado. *Arct. Ant. Alp. Res.* **42**, 198-209 (2010).

36 Mills, H. H. Some implications of sediment studies for glacial erosion on Mount Rainier, Washington. *Northwest Sci.* **53**, 190-199 (1979).

37 Hodge, S. M. Variations in the sliding of a temperate glacier. *J. Glac.* **13**, 349-369 (1974).

38 Sanders, J.W., Cuffey, K.M., MacGregor, K.R. & Collins, B.D. The sediment budget of an alpine cirque. *GSA Bull.* **125**, 229-248, (2013).

39 Sanders, J.W., Cuffey, K.M., MacGregor, K.R., Kavanaugh, J.L. and Dow, C.F. Dynamics of an alpine cirque glacier. *Am. J. Sci*. **310**, 753-773 (2010).

40 Aðalgeirsdóttir, G. *et al.* Modelling the 20th and 21st century evolution of Hoffellsjökull glacier, SE-Vatnajökull, Iceland. *Cryosphere* **5**, 961-975 (2011).

41 Mackintosh, A., 2000. Glacier fluctuations and climatic change in Iceland. Unpublished PhD Thesis, University of Edinburgh.

42 Van Wychen, W. *et al.* Glacier velocities and dynamic ice discharge from the Queen Elizabeth Islands, Nunavut, Canada. *Geophys. Res. Lett.* **41**, 484-490 (2014).

43 Hicks, D. M., McSaveney, M. J. & Chinn, T. J. H. Sedimentation in Proglacial Ivory Lake, Southern Alps, New Zealand. *Arct. Alp. Res.* **22**, 26-42 (1990).

44 Cuffey, K. M. et al. Entrainment at cold glacier beds. *Geology* **28**, 351-354 (2000).

45 Cuffey, K.M., Conway, H., Hallet, B., Gades, A.M. and Raymond, C.F. Interfacial water in polar glaciers and glacier sliding at− 17 C. *Geophys. Res. Lett.*, **26**, 751-754 (1999).

46 Herman, F. *et al.* Erosion by an Alpine glacier. *Science* **350**, 193 (2015).

47 Koppes, M. *et al.* Observed latitudinal variations in erosion as a function of glacier dynamics. *Nature* **526**, 100 (2015).
